# Evaluating the Role of Unit Cell Multiplicity in the Acoustic Response of Phononic Crystals Using Laser-Plasma Sound Sources

**DOI:** 10.3390/ma18061251

**Published:** 2025-03-12

**Authors:** Emmanouil Kaniolakis Kaloudis, Konstantinos Kaleris, Nikos Aravantinos-Zafiris, Michael Sigalas, Dionysios T. G. Katerelos, Vasilis Dimitriou, Makis Bakarezos, Michael Tatarakis, Nektarios A. Papadogiannis

**Affiliations:** 1 Institute of Plasma Physics and Lasers (IPPL), Hellenic Mediterranean University, Tria Monastiria, GR-74100 Rethymnon, Greece; mkaniolakis@hmu.gr (E.K.K.); dimvasi@hmu.gr (V.D.); bakarezos@hmu.gr (M.B.); mictat@hmu.gr (M.T.); npapadogiannis@hmu.gr (N.A.P.); 2 Physical Acoustics and Optoacoustics Laboratory, Department of Music Technology and Acoustics, Hellenic Mediterranean University, GR-74100 Rethymnon, Greece; 3 Department of Environment, Ionian University, GR-29100 Zakynthos, Greece; naravadinos@ionio.gr; 4 Department of Materials Science, University of Patras, GR-26500 Patras, Greece; sigalas@upatras.gr; 5 Department of Audio & Visual Arts, Ionian University, GR-49100 Corfu, Greece; dkaterelos@ionio.gr; 6 Department of Electronic Engineering, Hellenic Mediterranean University, GR-73133 Chania, Greece

**Keywords:** acoustic metamaterials, phononic crystals, laser-plasma sound sources, acoustic characterization

## Abstract

Acoustic metamaterials and phononic crystals are progressively consolidating as an important technology that is expected to significantly impact the science and industry of acoustics in the coming years. In this work, the impact of unit cell multiplicity on the spectral features of the acoustic response of phononic crystals is systematically studied using the recently demonstrated laser-plasma sound source characterization method. Specifically, by exploiting the advantages of this method, the impact of the number of repeated unit cells on the depth of the phononic band gaps and the passband spectral features across the entire audible range is demonstrated. These experimental findings are supported by specially developed computational simulations accounting for the precise structural characteristics of the studied phononic crystals and are analysed to provide a phenomenological understanding of the underlying physical mechanism. It is shown that by increasing the unit cell multiplicity, the bandgaps deepen and the number of resonant peaks in the crystal transmission zones increases. The resonant mode shapes are computationally investigated and interpreted in terms of spherical harmonics. This study highlights the tunability and design flexibility of acoustic components using phononic crystals, opening new paths towards applications in the fields of sound control and noise insulation.

## 1. Introduction

Acoustic metamaterials (AMs) and phononic crystals (PCs) are engineered materials that manipulate sound in ways that are not possible with conventional acoustic materials. Particularly, AMs exploit structures shorter than the wavelength of the sound they control to achieve unconventional behaviour like negative refraction and acoustic cloaking [1,2]. Conversely, PCs are artificial periodic arrangements of multiple components (unit cells) that affect acoustic wave propagation due to Bragg scattering. Each unit cell features an internal structure with regions of differing acoustic properties, such as acoustic impedance and/or sound speed [3,4,5]. Interaction between propagating acoustic waves and PCs results in a characteristic frequency response consisting of forbidden and permitted spectral regions, named phononic band gaps (stopbands) and transmission bands (passbands), respectively [6]. The acoustic spectral profile of PC is related to Bragg scattering [7,8,9,10], which takes place when the wavelength of the propagating acoustic wave (AW) is comparable to the features and periodicity of the structure [11]. Specifically, when the AWs propagate through the structure, they encounter regions of different acoustic impedances, forming interfaces upon which the AWs scatter. Superposition of the scattered waves from multiple layers gives rise to interference patterns. When the Bragg condition for constructive interference is satisfied, wave propagation is allowed, and the passband regions are formed. Conversely, for the frequencies satisfying the destructive interference condition, acoustic transmission is prohibited, leading to the emergence of spectral bandgaps.

Importantly, the passbands and stopbands of PCs can be precisely engineered by designing the geometrical and structural characteristics of the unit cells. Their capability to tailor their acoustic response makes PCs ideal for various applications. Particularly, PCs are utilized in noise insulation [12,13,14], vibration isolation [15,16], sound attenuation [17] and soundproofing [18], where the targeted attenuation of desired frequencies is achieved by exploiting phononic crystal bandgaps. Additionally, control of the passband features facilitates the design of acoustic waveguides [19,20,21,22,23,24] and acoustic filters [5,11,25,26,27], enabling the selective transmission of AWs by amplifying the transmission in specific frequencies. Finally, apart from precisely tailored acoustic filtering [21], specially designed PCs can bend, focus or redirect the propagating sound waves [28,29,30], rendering them useful in applications such as acoustic cloaking and directional sound generation [31,32].

The complexity of acoustic wave-structure interaction in PCs often renders the comprehension and analytical description of their behavior difficult. Currently, the design of new PCs is commonly done via complex numerical simulations and is often based on the experience of the designer rather than on a systematic approach. Moreover, experimental evaluation of developed structures on the level of a single or a few unit cells has proved difficult with conventional methods. The recently demonstrated Laser-Plasma Sound Source (LPSS) method [33] allows for evaluation of acoustic features of the PC transmission spectrum, including band gap depth and resonant features, with unprecedented accuracy. This work exploits the LPSS method to identify the impact of the number of unit cells on the transmission spectra of PCs across the entire audible range. Particularly, we experimentally evaluate the transmission spectra of linear PCs with different lengths, developed via repetition of the same unit cell. LPSSs are highly effective in characterizing PCs due to their massless and point-like geometry, rapid pressure profile, broad frequency spectrum and high acoustic energy [33]. Particularly, we focus on the impact of the number of unit cells on the following: (a) the depth of the bandgaps; and (b) the profile of the resonant features in the passbands of the acoustic transmission spectrum within the entire audible range. Our findings reveal a clear connection between the number of unit cells and the depth of the gaps. Specifically, the depth increases with the increasing number of unit cells. Furthermore, an increase in the cell multiplicity leads to an increase in the number of resonant peaks within the second and third passbands. The experimental observations are validated by specially developed computational simulations, showing excellent agreement. This work reveals the exact role of the structural multiplicity in the shaping and control of the spectral features of PCs. More specifically, it provides an experimental methodology for the determination of the number of unit cells required to achieve a specific bandgap depth, especially with respect to the behavior of a structure with infinite length. It also demonstrates the impact of cell multiplicity on the resonant features of the passbands, which can be directly related to the spectral flatness of the transmission regions. To the best of our knowledge, there are no publications that provide computationally validated experimental evidence on these aspects. This is potentially due to the prior absence of sufficiently accurate experimental methods for their investigation, a shortcoming that is here overcome by the LPSS method. In this respect, the study contributes to the systematization of the design and optimization process of PCs. More generally, it can lead to efficient PC designs with tailored control over AW transmission, which can be useful in various scientific and industrial applications, especially in room acoustics and noise control.

## 2. Materials and Methods

**Figure 1:**
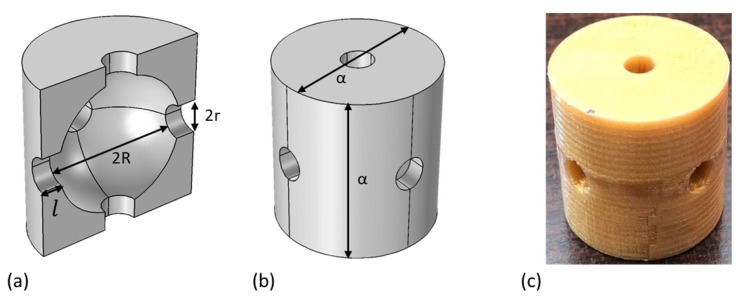
Internal (**a**) and external (**b**) design of the unit cell, where a=29 mm is the lattice constant, R=0.395a is the sphere radious, r=0.095a  is the conduit radious and l=0.105α is the conduit length. (**c**) Image of a 3D-printed unit cell made of PLA.

The phononic crystals used in this study were developed via the repetition of a cylindrical unit cell consisting of a spherical air cavity surrounded by six air conduits. The samples were fabricated in house using polylactic acid (PLA) (XYZprinting), with the fused filament technique on an 3D printer (XYZprinting da Vinci Super, New Taipei Taiwan). The unit cells had a lattice constant a=29 mm, with a sphere radius of R=0.395a, a conduit radius of r=0.095a and an infill of 100%. The infill value represents the porosity of the crystal, with an infill of 100% corresponding to a porosity of 0%. Four structures were fabricated with the repetition of 2, 3, 4 and 5 unit cells (Figure 1). This structure has already been shown, both experimentally and numerically, that provides wide and complete acoustic band gaps [9,33].

Evaluation of the impact of the unit cell multiplicity on the transmission spectrum of the PCs was carried out by the use of laser plasma sound sources. LPSSs are generated in ambient air by the thermoelastic reaction of a localized air volume following Laser-Induced Breakdown (LIB). High-intensity, short or ultrashort laser pulses ionize the air particles, generating high-temperature free electrons via multiphoton ionization and inverse Bremsstrahlung. These hot electrons thermalize the heavier ions via various physical processes, e.g., recombinations, causing the rapid thermal expansion of the air volume and the formation of a characteristic acoustic N-pulse [33]. The laser generated acoustic N-pulses exhibit a first order high-pass profile at the lower end of the acoustic spectrum. For excitation from nanosecond laser pulses, this first order profile spans from the infrasounds (<20 Hz) to the high end of the audible spectrum (~20 kHz) or even the near ultrasounds (~60 kHz). Peak pressure levels can exceed 130 dB, depending on the optical energy deposited by the laser pulses in the excitation volume. Extensive studies of the N-pulses and their relation to the laser pulse characteristics and parameters can be found in [34,35,36,37,38].

**Figure 2:**
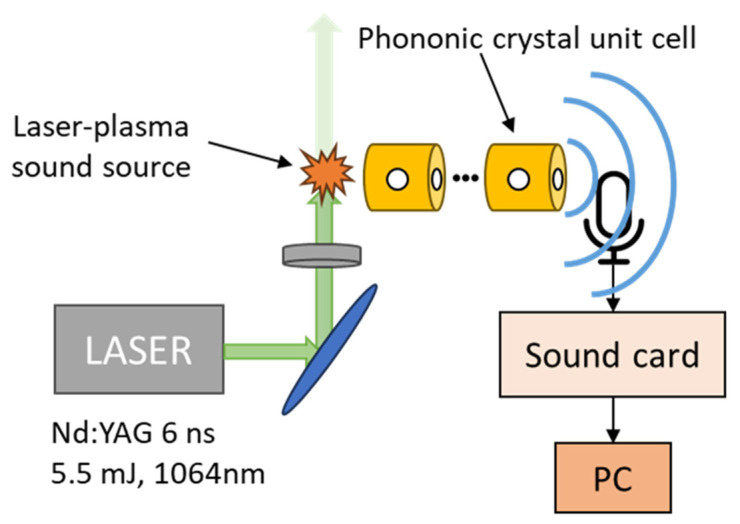
Schematic diagram of the experimental setup used for the acoustic evaluation of phononic crystals via laser-plasma sound source excitation.

In our experiments, LPSS formation was done by a 6 nanosecond Nd: YAG pulsed laser system (Quantel Brilliant B) with 1064 nm central wavelength and up to 10 Hz repetition rate. Here, a repetition rate of 5 Hz was used that allows for a 200 ms time interval between consecutive pulses. The laser beam was focused with a 7.5 cm bi-convex lens in the ambient air. The laser pulse energy of 5.5 mJ ensured that the laser-plasma source was perfectly spherical and hence omnidirectional, as evaluated by fast shadowgraphy. The LPSSs were positioned ~2 mm from the input hole of the structure. The acoustic measurement system was based on a broad frequency range (90 kHz) and high dynamic range (35 dB(A)–160 dB) microphone (G.R.A.S 46BE and power module 12AK). A sound card (RME Fireface 802) with a 192 kHz sampling rate and 24-bit resolution was used for the sampling of the acoustic signals. The microphone was placed at a distance of ~3 mm from the output hole, so that it was sufficiently close to the structure but without blocking the output hole. The experimental set up is shown in Figure 2. The acoustic signals were recorded with the Audacity software (version 3.7.0) [39]. Each measurement contained a train of 120 consecutive responses, corresponding to a total duration of 24 s. Noise reduction was achieved by taking the average waveform of the 120 acoustic responses. An analytical description of the signal processing methodology can be found in [33].

Finally, numerical simulations regarding the PCs’ acoustic response were performed using COMSOL Multiphysics^®^ (version 5.5) and particularly the Acoustics Module and the Solid Mechanics interface. The Finite Element Method included in the commercial package was used to numerically solve the wave propagation following equation for sound:

(1) $$\nabla \left(-\frac{1}{\rho_{c}}\left(\nabla p_{t}-q_{d}\right)\right)-\frac{k_{eq}^{2}p_{t}}{\rho_{c}}=\frac{4\pi }{\rho_{c}}S\delta (x-x_{0})$$

(2) $$S={\omega e}^{i\varphi }\sqrt{\frac{\rho_{c}u_{air}p_{rms}}{2\pi }}$$

where $p_{t}$ is the total acoustic pressure, $\rho_{c}$ is the density of ambient air, $k_{eq}=\frac{2\pi }{\omega }$ is the wavenumber and ω the angular frequency, $q_{d}$ is the dipole domain source and $\delta (x-x_{0})$ is the Dirac’s delta function. Additionally, S is the amplitude of the monopole point source, $p_{rms}$ is the reference pressure and $u_{air}$ is the speed of sound in ambient air. The source spectrum exhibits a 1st order high pass profile (S~ω) in order to emulate the LPSS spectrum within the frequency range of interest.

For the numerical calculations, the following values for the properties of the PLA were used: Poisson ratio v=0.35, Young’s Modulus E=3.53 GPa and density $\rho =1240\;kgm^{-3}$. The acoustic-solid interaction frequency domain interface was used in the model for the coupling of the ambient air with the solid boundary. A monopole point source of flow type was used to represent the laser-plasma sound source within the audible frequency range. Perfectly Matched Layers (PML) were used at the boundaries of the computational domain to eliminate boundary reflections. The transmission spectra are calculated according to the following equation:

(3) $$T=20{log}_{10}\frac{p_{T}}{p_{I}}$$

where $p_{T}$ and $p_{I}$ represent the transmitted and incident pressure field on the two sides of the sample.

## 3. Results

**Figure 3:**
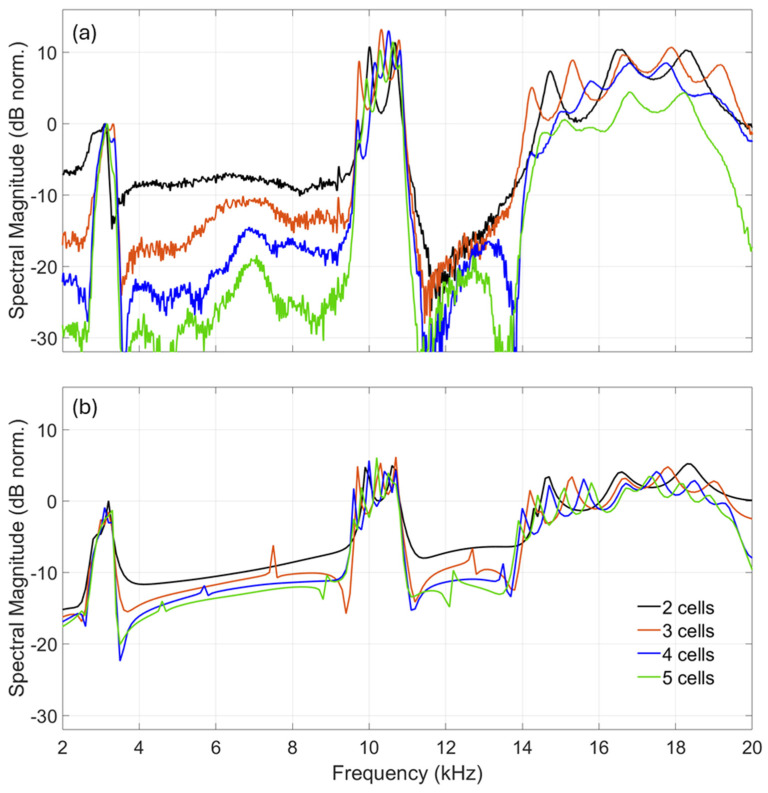
Experimentally measured (**a**) and simulated (**b**) acoustic transmission responses of phononic crystals consisting of 2–5 unit cells to laser-plasma sound source excitation.

Figure 3a,b show the measured and simulated spectral responses of the PCs to LPSS excitation in the frequency range from 1.5 to 20 kHz. The laser-plasma source was placed at a distance of ~2 mm from the input hole, resulting in acoustic wave propagation along the depth of the PC. The experimental results shown in Figure 3a clearly reveals two phononic bandgaps between 3.6 and 9.4 kHz and between 11 and 13.6 kHz, as well as three passbands with characteristic resonant peaks at approximately 2.5–3.5 kHz, 9.5–11 kHz and 13.5–19.5 kHz. As expected, the frequency ranges of both the phononic bandgaps and the passbands remained approximately the same, independently from the number of unit cells. However, the phononic bandgaps became deeper with increases in the number of unit cells, as the structure’s behaviour tends towards that of the infinite structure.

To quantify the deepening of the band gap, the energy ratio $E_{R}$ is calculated as follows:

(4) $$E_{R}=E_{bg1}^{\left(n\right)}/E_{bg1}^{\left(2\right)}$$

$E_{bg1}^{\left(n\right)}$ is the energy in the 1st band gap of an n-cell structure and is given by the following:

(5) $$E_{bg1}^{\left(n\right)}=\sum_{f_{low}}^{f_{up}}S^{2}(f)$$

where $f_{low}=3850\;\mathrm{Hz},\;f_{up}=9050\;\mathrm{Hz}$ denote the lower and upper frequencies of the band gap and $S^{2}(f)$ is the spectral energy obtained by fast Fourier transform (FFT) of the transmission signal $s_{T}(t)$, expressible as the following:

(6) $$S^{2}\left(f\right)={\left(FFT\left\{s_{T}\left(t\right)\right\}\right)}^{2}$$

**Figure 4:**
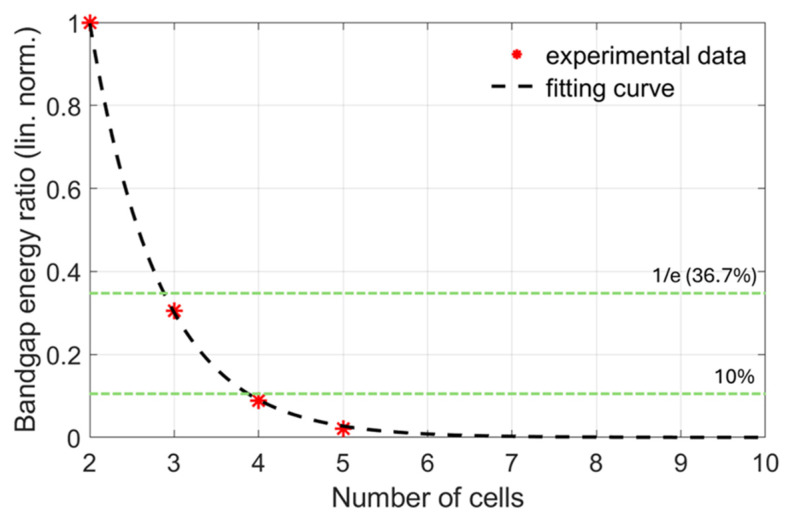
Decay of the energy of the first band gap with increasing cell multiplicity. The horizontal green lines mark the 1/e (36.7%) and 10% energy drop criteria, with respect to the 2-cell structure.

In $E_{R}$, the energy of the n-cell structure is compared to that of the 2-cell structure $\left(E_{bg1}^{\left(2\right)}\right)$, which is considered to be the PC with the lowest cell multiplicity. The energy ratio taken by the experimental measurements can be seen in the scatter plot of Figure 4 (experimental data). One step further, $E_{R}$ can be used for the estimation of the sufficient number of unit cells for the PC to approximately function as an infinite phononic crystal. For this purpose, we have adopted the criterion of band gap energy drop by a factor of 1/e (~36.7%), expressible as the following:

(7) $$\frac{E_{bg1}^{\left(n\right)}}{E_{bg1}^{\left(2\right)}}\leq \frac{1}{e}\;\Rightarrow 10\log_{10}\left(\frac{E_{bg1}^{\left(n\right)}}{E_{bg1}^{\left(2\right)}}\right)\leq -4.34\;dB$$

where e is the base of the natural logarithm.

The resulting 4-points graph is fitted by an exponential decay function, expressible as the following:

(8) $$g\left(n\right)=a\exp\left(-bn\right)$$

with a=11, b=1.2, which was found to perfectly match the observed bandgap energy decay. The results are presented in Figure 4 (see below), showing that 3-cell structures already fulfill the 1/e energy drop criterion. Even for a stricter criterion of 10% (−10 dB), 4 cells are sufficient.

**Figure 5:**
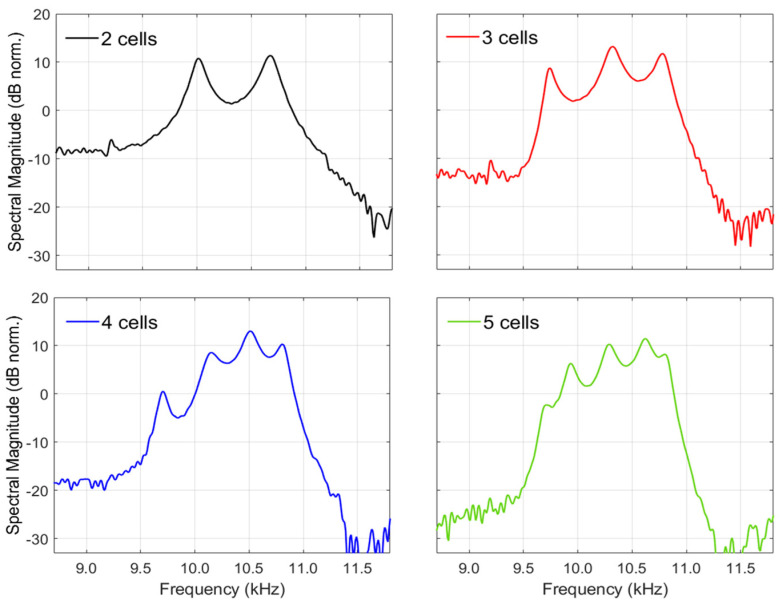
Details of the measured spectral profiles shown in Figure 3a for the frequency range 8.5–12 kHz. The resonant peaks of the second passband are equal in number with the number of the unit cells.

Moreover, these experimental results reveal the impact of cell multiplicity on the passband frequency regions. Particularly, the first passband (~2.5–3.5 kHz) appears as a mainly uniform and broadened spectral feature. Distinct spectral peaks can be vaguely observed in some of the responses. In the second passband, frequency range (~9.5–10.9 kHz), distinct resonant peaks are clearly observed which are equal in number to the unit cells of the structure, as shown in Figure 5. In the third passband (~13.5–19.5 kHz), the behaviour of the resonant peaks is more complex, where the number of peaks N appears to relate to the unit cell number n according to the formula N=2n−1. Notably, the experimental findings of Figure 5 show that by increasing the number of unit cells, the spectral flatness of the passband regions increases. Here, as a measure of spectral flatness, the average peak-to-dip distance is taken, which approximately is 10 dB for the 2-cell structure while it reduces to ~3 dB in the 5-cell structure. This is because the contribution of the increased number of resonant peaks leads to spectral overlaps that shape a flat spectrum. It should be mentioned here that the high-pass slope observed in the passband regions, as for example in the 4-cell and 5-cell structures, can be attributed to the high pass profile of the LPSS excitation and is not an inherent acoustic feature of the structure. Hence, the slope would be absent in the structure response to a flat acoustic source. A thorough interpretation of the physical mechanisms leading to the formation of these features is given in the Discussion.

Furthermore Figure 3b demonstrates the excellent agreement between the computational model and the experimental results in terms of the relation of the bandgap depth and the number of resonant peaks of the passband regions. Minor deviations can be attributed to the fact that the computational model simulates the receiver as a point with negligible dimensions in contrast to the real microphone, which effectively is a surface. On the microphone, the more complex superposition of wave fronts exiting the PC leads to deeper bandgaps, especially as the number of unit cells increases.

## 4. Discussion

**Figure 6:**
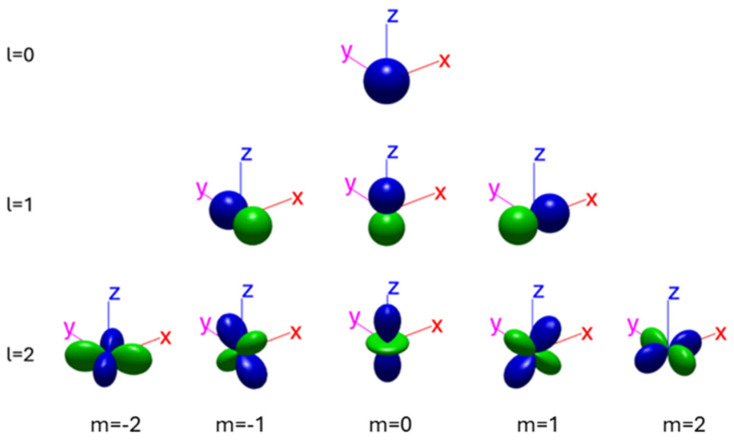
Geometrical shapes of spherical harmonics for l=0, 1, 2 and m=0, ±1, ±2, taken from [41]. The different colors indicate opposite phase of the acoustic pressure.

Here we present an analysis of the formation of the transmission spectral peaks from the interaction of the propagating acoustic wave and the cavities and conduits forming the internal structure of the PCs. Specifically, the acoustic impedance, or equivalently the acoustic refractive index of the air encountered by the propagating acoustic waves, alternate successively between the narrow cylindrical conduits and the comparatively wider spheres within the PC structure. As a result, the acoustic behavior of the PCs resembles that of a layered material, where sound waves at each interface between successive layers are partially reflected, in a process resembling Bragg scattering in crystals. The reflected waves then interfere with each other constructively (in phase) or destructively (out of phase) forming spectral regions where propagation is enhanced or suppressed respectively. In the passband regions, Helmholtz resonances appear in the spherical cavities while standing waves appear in the cylindrical conduits, especially in the high end of the audible range. The resonances in the spherical cavities can be estimated by the spherical harmonic functions $Y_{l}^{m}(\theta ,\varphi )$, which are given by the following equation [40]:

(9) $${𝛻}^{2}Y_{l}^{m}\left(\theta ,\varphi \right)+l\left(l+1\right)Y_{l}^{m}\left(\theta ,\varphi \right)=0$$

where θ and φ represent the azimuth and elevation angles, respectively, while l and m represent the degree and order of the harmonic function, respectively. A schematic representation of the pressure distribution inside a spherical cavity for the first three degrees (l=0, 1 and 2) of the spherical harmonic is shown in Figure 6. The different colours in the geometrical distributions represent out of phase pressure profiles.

**Figure 7:**
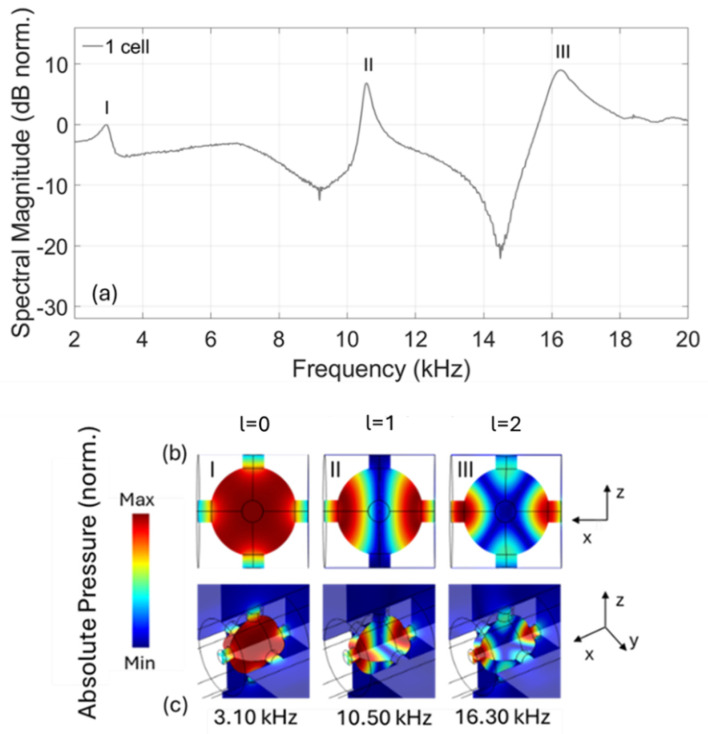
(**a**) Experimentally measured transmission spectrum of the unit cell, with I, II and III indices denoting the l=0, l=1 and l=2 resonances, respectively. (**b**) 2D and (**c**) pseudo-3D representations of the absolute pressure distribution (mode shapes) for the three resonant frequencies and l=0, 1, 2 denote the first three degrees of the spherical harmonics.

Figure 7a shows the transmission spectrum for the unit cell, where three resonant peaks appear with central frequencies of 3.1 kHz, 10.5 kHz and 16.3 kHz. In accordance with the simulated mode shapes (Figure 7b), namely the simulated pressure distribution at a particular frequency, these spectral peaks can be attributed to the spherical harmonics arising in the spherical cavity. Particularly, the first peak (I) corresponds to the zero-degree spherical harmonic with l=0, while the second (II) and third peaks (III) correspond to the first (l=1) and second (l=2) degree spherical harmonics respectively. The symmetry conditions of the studied scenario are dictated by the source, which is placed before the left hole of the structure, leading to an acoustic wave that initially propagates along the x axis (see Figure 7c). This results in a symmetric behaviour of the structure in the y and z directions, as can be observed by the two-plane representations of the figure (Figure 7c). It should be noted that the mode shapes shown in Figure 7b,c represent absolute pressure. Hence, they do not contain any phase information, in contrast to the graph of the spherical harmonics (Figure 6), in which the opposite phase is denoted with different colours.

The fundamental resonance frequency of a spherical cavity with six narrow conduits can be calculated from the following equation [42]:

(10) $$f_{0}=\frac{u_{air}}{2\pi }\sqrt{\frac{6A_{c}}{V_{c}L_{c,eff}}}$$

(11) $$L_{c,eff}=L_{c}+1.7r$$

where $A_{c}=\pi r^{2}$ is the conduit cross sectional area, $u_{air}$ is the speed of sound in ambient air, $V_{c}$ is the volume of the spherical cavity, $L_{c}$ is the conduit length, $L_{c,eff}$ is the effective conduit length and r the conduit diameter. For the geometry of the unit cell under investigation, the fundamental frequency is $f_{0}=2.85\;kHz$, which is in accordance with the experimentally measured ($f_{0}\sim 3\;kHz$) and numerically calculated frequency.

**Figure 8:**
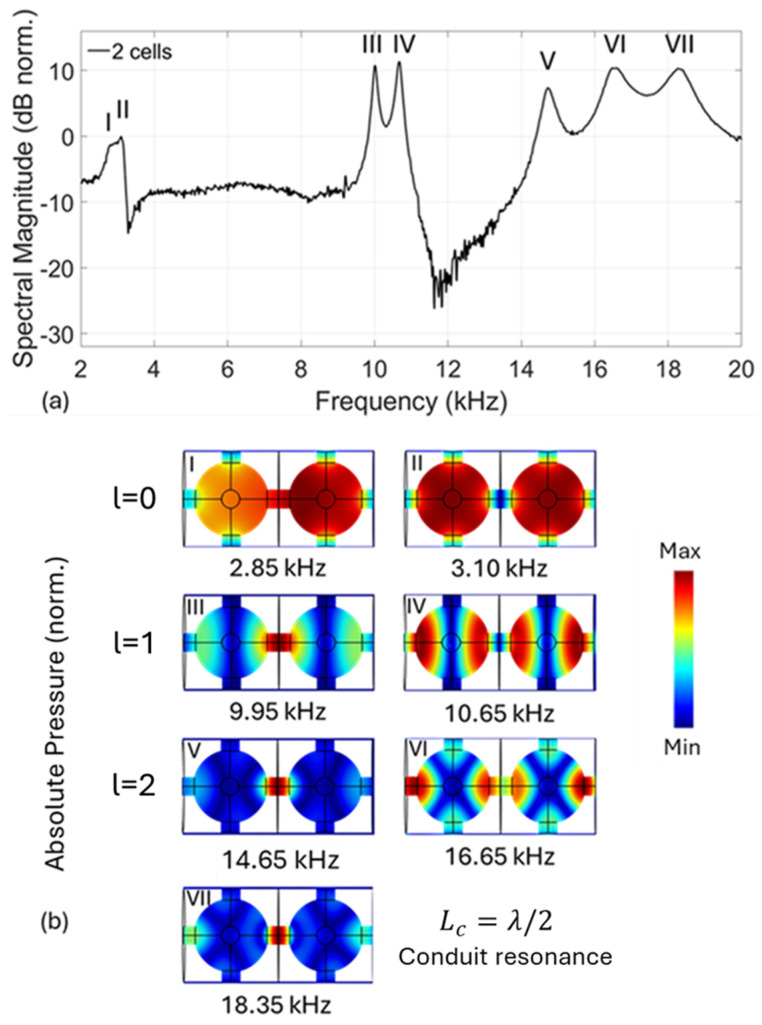
(**a**) Experimentally, measured transmission spectrum of the 2-cell structure, with I, II and III indices denoting the resonance peaks of the first passband. The III and IV indices denote the resonance peaks of the second passband and the V, VI and VII indices denote the resonance peaks of the third passband. (**b**) 2D representation of the absolute pressure distribution (mode shapes), l=0, 1 and 2 denote the first three degrees of the spherical harmonics, and λ is the longest wavelength for which the standing wave condition in the conduit is fulfilled.

Regarding the 2-cell PC, the transmission spectrum shown in Figure 8a features three passband regions, while Figure 8b corresponds to the simulated mode shapes. Here, the first passband features two resonant peaks (I and II), while the second passband feature two prominent resonant peaks (III and IV) and the third features three (V, VI and VII). The appearance of additional resonances can be attributed to the coupling between the two cavities via a conduit with a length that is double the length of a single conduit. Particularly the resonant peaks of the first passband correspond to the symmetric and antisymmetric modes of the zero-degree spherical harmonic (*l* = 0) of the spherical cavities, coupled via the cylindrical conduit. The frequencies of the symmetric and antisymmetric modes exhibit a small frequency shift from the fundamental frequency $f_{0}$ due to the coupling factor. The same underlying mechanism also forms the two resonant peaks of the second passband, where the spherical harmonic forming the resonant peaks in this case is the first degree (l=1). In the third passband, the resonant peaks IV and V again correspond to the symmetric and antisymmetric modes of the second degree harmonic (l=2). The third resonant peak with experimentally measured frequency, 18.35 kHz, however, can be attributed to the conduit itself acting as a resonator. Particularly, this conduit resonance corresponds of the λ/2 standing wave condition, taking into account the length and the end correction for the conduit geometry. Calculation of the conduit resonant frequency using the Equation (7) gives $f_{c}=18.3\mathrm{kHz}$, expressible as the following:

(12) $$f_{c}=2\frac{u_{air}}{L_{c,eff}}$$

For a larger number of unit cells, the number of resonant peaks within the first and second passbands are equal to the unit cell multiplicity, with each peak associated with symmetric and antisymmetric modes, respectively. However, due to the very small Δf for the harmonic of the first degree, the peaks unify, forming a single and widened peak. Finally, for the third passband, the number of resonant peaks follows the relation N=2n−1. In this relation, n resonances pertain to the spheres acting as resonators, with their symmetric and antisymmetric modes, which are equal to the number of the spheres. The additional resonances originate from the n−1 conduits acting as resonators. The conduits also constitute a system of interconnected resonators, having symmetric and antisymmetric modes. Since the number of modes equal the number of conduits, they are n−1. By adding the sphere and conduit modes, we get the total 2n−1 resonant peaks observed in the 3rd passband both in the measurements and simulations.

## 5. Conclusions

In this work we studied the impact of the multiplicity of unit cells on the profile of the acoustic response of PCs. We presented our experimental results and simulations demonstrating that by varying the number of unit cells it is possible to manipulate the PC spectral profiles. It was shown that an increase in the multiplicity of unit cells results in an increase in the depth of the first and second bandgap regions. Specifically, the acoustic energy in the first band gap decays exponentially with the number of cells. Based on this finding, a methodology to estimate the number of unit cells required for the structure to approximate the behavior of an infinite structure was outlined. Furthermore, it was shown that cell multiplicity also determines the number of resonant peaks in the passband regions. With the help of computationally evaluated mode shapes, the resonant peaks were attributed to distinct spherical harmonics of the spherical cavities and standing wave modes of the cylindrical conduits. This relation was found to be in accordance with calculations of the resonant frequencies using the analytical formulas for the spherical harmonics and standing waves. Notably, it was shown that an increase in the unit cell multiplicity leads to an increased spectral flatness of the passband region. These results provide a clear demonstration of the controllability of the acoustic response of PCs by modulating their unit cell multiplicity. They allow for the determination of the number of unit cells in order to achieve the desired (a) acoustic energy suppression in the bandgaps and (b) spectral flatness in the passband regions. Moreover, they contribute to the understanding of the functionality of such PC structures as well as to the systematization of their design process. The control of the acoustic wave transmission via PCs paves the way for innovative applications in the field of acoustics, such as noise insulation and room acoustics.

In the future, the presented methodology will be applied to other PC structures, including structures with defected cells and higher-order structures. The results will be compared to the findings presented here, to identify potential global patterns in the behavior of PCs. Also, an investigation will be carried out to identify the impact of the material density of the structure on the observed transmission spectra. This will be achieved by measuring identical structures that are 3D printed with varying infill densities of the PLA material via the LPSS method.

## Data Availability

The original contributions presented in the study are included in the article. Further inquiries can be directed to the corresponding author.
